# The Recognition and Management of Adverse Effects in Electroconvulsive Therapy: Findings From a Finnish Survey Study

**DOI:** 10.1002/brb3.70227

**Published:** 2025-01-19

**Authors:** Saara H. Huoponen, Katrin Sisa, Tom Saari, Markku Taittonen, Ulla Ahlmén‐Laiho

**Affiliations:** ^1^ Turku University Central Hospital, Division of Operative Services, Intensive Care and Acute Pain Management Turku Finland; ^2^ Turku University, Feculty of Medicine and Turku University Central Hospital, Division of Operative Services, Intensive Care and Acute Pain Management Turku Finland

**Keywords:** adverse effects, attitudes, confusion, electroconvulsive therapy, nausea, pain, recognition

## Abstract

**Aim:**

The aim of the study was to survey the observed incidence of adverse effects (AEs) related to electroconvulsive therapy (ECT) in Finnish neuromodulation units, as well as to explore what medical interventions are used to prevent and treat them in those units.

**Methods:**

An electronic survey was conducted among Finnish neuromodulation units at the end of 2022. The survey included 35 questions related to AEs and their prevention and/or treatment in the responding units’ ECT patient populations.

**Results:**

Our survey reached 19 out of 26 units in Finland, with 17 units completing the full questionnaire. Headache, myalgia and postictal confusion (PIC) emerged as the most frequently reported AEs. Nausea and high blood pressure were reported less often. Only a few units reported AEs known to be rare, such as accidental awareness during general anesthesia and the aspiration of gastric contents.

However, there was considerable variation in the recognition and treatment of those ECT‐related AEs the diagnosis of which depends more on patients’ self‐reporting, including headache, myalgia or nausea. Five units (29%) reported frequent or occasional headache or myalgia and four units (24%) reported occasional nausea experienced by their patients, but these AEs were addressed pharmacologically in those units neither by prophylaxis nor by treatment. This raises concern about whether these AEs are perceived as an insignificant issue in delivering ECT treatment, thus requiring no intervention, or if those AEs should be better recognized and and managed more actively.

**Conclusions:**

AEs related to ECT treatment are common, but some still appear poorly recognized and treated. Regarding treatment adherence, minimizing potential AEs whenever feasible can be considered important. A thorough preoperative assessment of patients is required to identify possible risk factors for AEs. An objective and structured evaluation tool for recognizing adverse effects in patients undergoing ECT treatment would be useful.

## Graphical abstract

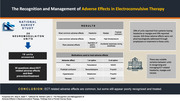

Significant Outcomes: (1) The adverse effects of ECT treatment are common, with headache and confusion being the most frequently reported. (2) The recognition of those AEs, which depend on patient self‐reporting, such as headache and nausea need to be better identified so that they could be addressed pharmacologically. (3) Objective tools should be developed for identifying AEs in ECT patients, and awareness of the existence of these AEs should be increased among ECT providers so that they can be effectively managed.

Limitations: (1) Our survey did not receive answers from some of the smaller neuromodulation units during the survey outreach, resulting in a 69% national response rate. (2) Since the study was conducted as a survey as opposed to a prospective observational one, its responses contain subjectivity. To determine precise absolute incidences of adverse effects, a comprehensive prospective randomized study should be conducted in the future.

## 1 Introduction

Electroconvulsive therapy (ECT) is an effective form of treatment for severe and treatment‐resistant depression (UK ECT Review Group 2003). It can be regarded as a first‐line options in situations requiring a prompt therapeutic response, such as when the patient is acutely suicidal or psychotic (Kellner et al. 2005; Fink and Taylor 2007).

While ECT has been proven safe and generally well tolerated, it has individually varying adverse effects (AEs). These effects tend to be temporary and can be mostly prevented or managed medically, which is reassuring (Karaaslan et al. 2019; Tzabazis et al. 2013). However, it is essential to avoid underestimating the significance of these adverse effects as they can substantially affect patients’ adherence to treatment regimens if perceived as overly burdensome or disruptive to their daily lives. Premature discontinuation of ECT treatment before achieving a clinical response poses a concern for patients at risk of suicide, with potentially fatal consequences.

According to prior literature, postictal confusion (PIC) is reported as one of the predominant AEs of ECT, affecting over 35% of patients (Ittasakul, Jarernrat, and Tor 2021). PIC can be alleviated pharmacologically, which should be utilized when PIC is accompanied by aggressiveness. Memory impairment is a common AE of ECT, particularly when the treatment is administered bilaterally. This memory loss can often be more persistent than other AEs. Anterograde amnesia results in patients recalling little from the ECT treatment period, while retrograde amnesia can lead to the loss of memories from before the treatment began and ECT can cause both types of amnesia. However, memory impairment is generally temporary and tends to improve over time (Sackeim et al. 2000).

Headache is another frequently observed adverse effect of ECT, with an estimated incidence exceeding 20% among ECT patients (Haghighi et al. 2016; Kertesz, Trabekin, and Vanetik 2015). Myalgia is also recognized as relatively common; Haghighi et al. (2016) reported an incidence of 9% in their study, while Dinwiddie, Huo, and Gottlieb (2010) presented a higher incidence, with over 30% of patients suffering from significant myalgia within 24 h after their first ECT session.

Post‐ECT nausea and vomiting (PENV) is also a well‐known AE of ECT treatment. However, there is currently limited research data available specifically on the incidence of PENV. In studies on surgical patients, the incidence of postoperative nausea and vomiting (PONV) after opioid‐free anesthesia, perhaps the closest surgical anesthesia equivalent to the methods used in ECT, varies between 10% and 24% (Beloeil et al. 2021; Massoth et al. 2021).

Other adverse effects include significant hemodynamic changes such as hypertension and arrhythmias observed in the electrocardiogram (ECG), accidental awareness during general anesthesia, prolonged seizures, and respiratory complications, including prolonged apnea and aspiration of gastric content (Andrade, Arumugham, and Thirthalli 2016). Serious adverse effects resulting in extended hospitalization or death, have been found to be rare (Kaster et al. 2021).

Currently, 26 neuromodulation units in Finland offer ECT treatment to psychiatric patients, all within the public healthcare system. A recently implemented national quality monitoring database has been established for Finnish ECT providers, but the database does not gather detailed information specifically related to anesthesia practices or the incidence of AEs. No comprehensive survey of Finnish ECT anesthesia practices and ECT related AEs has been conducted before. Therefore, at the end of 2022, our research group conducted such a survey of Finnish ECT anesthesia practices. Questions included, among others, the number of ECT treatments per week, the anesthetic medications used, and the staff's training background. A major part of the survey focused on the incidence, prevention, and treatment of AEs at different units.

In the following sections, we will discuss the survey results, providing data on the occurrence of AEs associated with ECT, and the preventive or treatment measures applied. We examine possible reasons for differences in reported AEs across units and assess their impact on patient care. By addressing these aspects, we aim to contribute practical insights to ECT treatment and patient compliance. Additionally, we will consider potential follow‐up measures to enhance the safety and effectiveness of ECT treatment as well as improve patient satisfaction, making it less likely for patients to want to withdraw from treatment.

## 2 Materials and methods

Webropol, a Finnish survey and research tool for creating online questionnaires, was utilized to create a survey form. A link to the survey form was sent via email to all 26 units providing ECT in Finland with an explanation regarding the contents and purposes of the study. At the 2‐week mark, a reminder‐email message about the open survey was sent to the recipients who had not yet filled out the form or responded in another manner.

The questionnaire contained 72 questions. The questions focused on the approximate weekly number of ECT treatments administered (categories: over 30, between 10 and 30, and less than 10) and available staff resources in the units. The next set of questions (27 in total) focused on the selection and use of anesthetic drugs and adjuvant medications for preventing and treating AEs. The third set of questions was dedicated to the monitoring methods of vital functions used during treatment.

The fourth set comprised eight questions on the incidence of various AEs. Additionally, we inquired separately whether the unit had implemented practices to prevent ECT treatment‐related AEs, such as aspiration of gastric contents. We focused the survey questions exclusively on short‐term AEs of ECT treatment, recognizing the potential for pharmacological interventions in managing these conditions. Therefore, we did not include inquiries about memory‐related AEs of ECT in the survey.

Participants were encouraged to provide feedback through an open‐text box after completing each section of questions. Respondents were also requested to specify their workplace unit at the end of the questionnaire. This step facilitated outreach to entities that had not responded yet. The survey answers were not connected to this identifying factor.

## 3 Results

Sixteen units completed the online form by the end of the initial 2‐week survey period. After the reminder‐email was sent to the units that had not yet responded, the final number of responding units increased to 18, resulting in a 69% response rate among all Finnish ECT units. One of these units, however, did not respond to questions regarding the incidence and treatment of AEs, and the percentages of these questions are therefore calculated based on responses from 17 units. Some smaller units offering ECT treatment only on an occasional basis to a very limited number of patients did not respond. One of these units emailed the principal investigator, indicating that they do administer ECT treatments but, citing the low frequency of fewer than one patient per week, they chose not to participate in the survey. In conclusion, contact was made with 19 out of the 26 units in Finland.

The unit sizes are categorized based on the number of ECT treatments carried out weekly. Eight units (45%) reported conducting more than 30 treatments per week, while nine units (50%) reported offering 10 to 30 treatments per week. One unit (5%) reported administering fewer than ten treatments per week.

The units were requested to assess how frequently their patients appeared to suffer from AEs typical of ECT treatment. Regarding pain stemming from various causes, the responses are as follows: three (18%) units reported headaches requiring medication to be frequent, and eight (47%) units reported them as an occasional problem among ECT patients. The remaining units reported less frequent instances of headaches. The most typical medication administered for both the prevention and postprocedural treatment of headache was either paracetamol (acetaminophen) or a nonsteroidal anti‐inflammatory drug (NSAID). Myalgia requiring treatment after the procedure was reported to occur frequently in one unit, occasionally in six (35%) and rarely in ten (59%) units. The incidence of injection‐associated pain caused by intravenous anesthetic was not specifically queried in the study, but regarding the use of lidocaine for treating this injection associated pain, eight (47%) units reported administering intravenous lidocaine as needed.

Post‐ECT nausea or vomiting (PENV) requiring medical intervention was occasionally reported in five (29%) units, rarely in ten (59%) units, and never in two (12%) units. The most typical group of drugs used in the prevention and treatment of PENV was 5‐HT3 receptor antagonists.

According to the survey, an increase in blood pressure requiring pharmacological intervention occurred frequently in one unit, occasionally in six (35%) units, rarely in nine (53%) units, and never in one unit. Hypertension was most typically treated with beta‐blockers, although clonidine, nitroglycerin, and propofol were also noted as alternative treatments.

PIC and associated aggression requiring pharmacological intervention were reported to occur occasionally in eight (47%) and rarely in nine units (53%). This AE was typically managed with benzodiazepines or propofol. Additionally, one unit mentioned using haloperidol to control confusion and aggression. Medications used to treat AEs in different units are listed in Table 1.

**TABLE 1:** Medications used to treat adverse effects.

Adverse effect	Medication used	% of units	Number of units
Post‐ECT nausea and vomiting	5‐HT3	76	13
	DHBP	29	5
	Corticosteroid	18	3
	Metoclopramide	18	3
Pain	Paracetamol	88	15
	NSAID	65	11
	Opioids	0	0
	Lidocaine	47	8
Hypertension	Beta‐blocker	76	13
	Clonidine	12	2
	Propofol	18	3
	Nitroglycerin	6	1
Postictal confusion	Benzodiazepine	88	15
	Propofol	76	13
	Haloperidol	6	1

Aspiration of gastric contents was reported by only two (12%) units, which is considered a very rare event. All other responding units stated that they had never encountered such a case. Another infrequently observed AE was accidental awareness during general anesthesia: 12 (71%) units reported it had never occurred, while four (24%) units had experienced rare occurrences. One unit reported that it was an occasional occurrence for patients to experience accidental awareness during the general anesthesia phase of an ECT treatment. Nine (53%) responding units had integrated a version of a so‐called checklist, often based on the original WHO surgical checklist, in their ECT protocol.

Five units (29%) reported rare or no occurrence of headache, myalgia or nausea requiring medication among their patients. Among these units, two could be considered large by Finnish standards (conducting over 30 treatments per week) while three were medium‐sized, conducting 10 to 30 treatments per week. This suggests that the weekly number of ECT sessions in these units ranges from 100 to 200.

The research results indicated that in Finland, some neuromodulation units never use any pharmacological intervention to prevent or treat PENV of their ECT patients. In terms of pain management, several units indicated that patients were only provided with paracetamol on an as‐needed basis for pain relief. Since propofol is known to cause injection pain (Jalota et al. 2011), we also combined the data of the main ECT anesthetic and lidocaine administration in the units. The responses revealed that in six (35%) units, primarily using propofol, patients' injection pain is never treated with lidocaine (Table 2).

**TABLE 2:** Percentage of units reporting never treating or giving prophylaxis for certain adverse effects.

Treatment of nausea	% of units	Number of units
Never prophylaxis	29	5
Never treatment	6	1
Never treatment or prophylaxis	18	3
Treatment of pain	% of units	Number of units
Never NSAID	35	6
Never lidocaine	53	9
Never NSAID or lidocaine	35	6
Mainly propofol and never lidocaine	35	6
Treatment of hypertension	% of units	Number of units
Never prophylaxis	29	5
Never treatment	6	1
Never treatment or prophylaxis	18	3
Treatment of postictal confusion	% of units	Number of units
Never treatment^*^	0	0

* Prophylaxis of postictal confusion was not part of the questionnaire.

We integrated the survey results regarding the reported incidence of AEs and the pharmacological interventions administered in the units. The findings indicate that, in certain units, despite acknowledging that patients experienced issues such as pain or nausea, these AEs are either not addressed pharmacologically at all or are treated inconsistently (Table 3).

**TABLE 3:** Nontreatment of adverse effects in units reporting them as frequent or occasional.

Nontreatment of adverse effects in units reporting them as frequent or occasional				
	Never prophylaxis		Never prophylaxis and only paracetamol	
Myalgia or headache	%	Units (*n*)	%	Units (*n*)
	29	5	24	4
PENV^*^	Never prophylaxis		Never treatment	
	%	Units (*n*)	%	Units (*n*)
	12	2	12	2

* No units reported PENV more than occasionally.

## 4 Discussion

### 4.1 ECT‐Related Pain

Our survey results revealed that headache was the most common AE, with 11 (65%) units indicating it as a frequent or occasional issue for their patients. This is in line with findings reported in prior studies (Karaaslan et al. 2019). Myalgia was reported to occur frequently or occasionally in seven (41%) units. Responding units typically treated pain associated with headaches or myalgia with paracetamol or NSAIDs. There is evidence suggesting that both paracetamol and NSAIDs alleviate tension‐type headaches equally effectively (Alnasser et al. 2023) and possibly are also equally effective when it comes to post‐ECT headache (Karaaslan et al. 2019). In the prevention of myalgia, on the other hand, there is evidence that NSAIDs are a more effective option (Karaaslan et al. 2019). The viability of using an NSAID depends naturally on the patient having no contraindications to it.

There is strong evidence indicating that lidocaine effectively reduces the injection pain caused by propofol (Euasobhon et al. 2016). The decision to avoid lidocaine in nine units may be influenced by propofol's demonstrated effect to shorten the ECT induced seizure duration (López‐Ilundain et al. 2023). Fortunately, injection pain can also be alleviated by cannulating an antecubital vein (Jalota et al. 2011). Interestingly, in recent studies, 5‐HT3 antagonists have shown to reduce propofol‐induced pain effectively (Bakhtiari, Mousavi, and Gharavi Fard 2021). Perhaps the benefits of 5‐HT3 antagonists in ECT should be considered in the future: in addition to preventing nausea, they can also alleviate the injection pain experienced by the patient.

### 4.2 Postictal Confusion (PIC)

Eight (47%) units reported occasional incidence of PIC requiring medication. PIC can pose significant challenges in ECT units due to the increased need for staff resources to manage agitated individuals in the recovery room, potentially endangering both the patient and staff. Moreover, the medications used to address confusion—commonly sedative benzodiazepines or propofol, as reported by the surveyed units—prolong the period during which patients need monitoring in the recovery room. Additionally, these medications may impair their cognitive functioning on the day of the treatment.

Previous studies have indicated that, if the patient has experienced PIC after ECT, it could be alleviated with dexmedetomidine during their subsequent treatment sessions (Feenstra et al. 2023). None of the surveyed neuromodulation units reported using dexmedetomidine even though the medication is widely used in Finnish hospitals in other contexts. Instead, propofol and benzodiazepines were most commonly used to treat PIC in Finnish ECT units. This raises questions about whether practices should be changed. The data mentioned above, which may well support dexmedetomidine as a drug‐of‐choice for post‐ECT agitation and confusion, should prompt discussion in the future among the Finnish ECT provider community.

### 4.3 Hypertension

Seven (41%) units reported that their patients experienced intervention‐requiring hypertension frequently or occasionally. The hemodynamic changes occurring during the ECT treatment, leading to a period of raised blood pressure, are well established. Blood pressure measured immediately after ECT treatment is typically elevated compared to the patient's baseline due to the hypertensive surge induced by ECT (Andrade and Bolwig 2014). However, it commonly decreases rapidly after the procedure. In some cases, blood pressure‐lowering medication may be necessary. According to our survey, beta‐blockers are the most commonly used drugs for managing post‐ECT hypertension in Finland.

Severely depressed patients often struggle to participate actively in the treatment of their somatic long‐term illnesses, which could result in poorly managed or undiagnosed hypertension. Significant changes in blood pressure and persistently elevated post‐ECT blood pressure prompt consideration of whether the patient has untreated hypertension. For reasons mentioned above, conducting a thorough preoperative evaluation of the somatic background and potential undiagnosed underlying diseases in patients undergoing ECT treatment is essential.

### 4.4 Post‐ECT Nausea and Vomiting (PENV)

Five (29%) respondents reported occasional occurrences of PENV requiring medication in their unit, while the rest reported even lower incidences. Based on the authors’ experiences in a large Finnish neuromodulation center, PENV appears to be quite common. As a result, a significant number of patients at our unit receive prophylactic treatment for it. However, according to the survey results, several units provided neither prophylaxis nor on‐demand medication to treat PENV to their patients. This discrepancy sparked our interest in finding out if the general anesthetic used could explain the difference in incidence of nausea. Propofol, the most common anesthetic in Finnish neuromodulation units (Huoponen et al. 2024), is known to reduce PONV risk in surgery patients (Apfel et al. 2004). The fact that the use of propofol is much less common at our unit than in many other Finnish units may contribute to the disparity in the nausea rates reported in the survey and our experiences. However, the data gathered does not allow verifying whether such a connection exists; further research is required.

### 4.5 Variation in Pharmacological Treatment and Recognition of Adverse Effects

The authors' experience is that headache, myalgia, and nausea are common AEs in ECT patients, an observation supported by prior research literature. A significant portion of patients in our unit receive pharmacological treatment for subjective AEs, usually preventively, such as antiemetic or anti‐inflammatory medication to prevent post‐ECT headache, myalgia, and nausea. Patients may not always report these AEs spontaneously but may mention, upon inquiry, experiences such as vomiting throughout the day following ECT treatment.

According to the findings of the national survey, although subjective AEs experienced by patients are acknowledged, they are not necessarily addressed with pharmacological treatment. Five units reported headaches or myalgia to be either frequent or occasional, yet their patients never receive preventive pain medication. In four of these units, the only analgesic given to patients was paracetamol as‐needed, despite there being prior data to suggest that NSAIDs may well be more effective, especially for myalgia. The same applied to PENV: four units reported that PENV occurred occasionally in their patient population, yet their patients did not receive any prophylaxis or treatment for it.

When comparing these results to the treatment of PIC, it's worth noting that no unit reported abstaining from administering pharmacological treatment for PIC. This also applies to units that reported PIC occurrences to be rare, not just to those where the phenomenon was reported as frequent or occasional. This is understandable: PIC‐associated aggression and agitation are easy to recognize and if a patient is confused and aggressive, withholding medication would also affect the staff's ability to work and could impact the care of other patients in the unit.

Recent studies indicate that healthcare professionals' understanding and attitudes regarding cancer pain and its treatment vary greatly, and there are significant deficiencies in pain management for patients (Galligan, Verity, and Briggs 2024; Makhlouf et al. 2020). This raises the question: do similar issues exist in the care of patients undergoing ECT? In addition to patients' potential challenges in expressing symptoms of AEs, it's essential to consider whether healthcare professionals may also have varied perceptions regarding these patients' experiences. Our survey results may also reflect the attitudes of healthcare providers: AEs might be perceived as an inherent aspect of treatment, potentially leading to oversight in the importance of preventing or alleviating them. Since effective and safe treatment exists for these AEs, we believe it should be offered to all who could benefit from it since AEs can cause patients to halt their treatment series.

Based on the results from our survey the perception of acceptable intensity of AEs differs among Finnish neuromodulation units. There seems to be significant variability in recognizing PENV or post‐ECT pain and thus being able to provide treatment and prevention for it, introducing a standardized scale for assessing these AEs, validated specifically for ECT patients, could be valuable.

### 4.6 Limitations

Finland has 26 neuromodulation units providing ECT, and our study reached 19 units, with 18 (69%) units responding. As mentioned earlier, we could not connect with some smaller units during the survey outreach. Likely, specific small units located in predominantly Swedish‐speaking communities might not have participated in the survey due to its availability only in Finnish. This could be attributed to the potential presence of staff in these units who may not be fluent in Finnish.

As the study was carried out in the form of a survey, its findings offer insights, but they cannot be viewed as definitive. For precise determination of, for example, adverse effect incidences, thorough prospective randomized research should be conducted in the future.

## 5 Conclusions

Adverse effects related to ECT treatment are prevalent, with headache, myalgia and PIC being the most common. The rarity of serious AEs (such as aspiration of gastric contents) according to our survey is reassuring.

There is substantial variability in how Finnish ECT providers recognize, prevent, and treat AEs. The study indicates that healthcare professionals may overlook or underestimate the subjective AEs experienced by ECT patients, potentially leading to inadequate pharmacological treatment. Severely depressed patients may have difficulty expressing symptoms of AEs, particularly early in a treatment series when their mental health is unstable. Efforts to recognize, prevent, and treat these AEs are crucial for patient satisfaction and promoting treatment adherence, particularly in the initial ECT sessions before favorable psychiatric effects manifest. Structured tools to measure AEs, modified for ECT patients, could be beneficial in recognizing subjective AEs. Potential AEs should be discussed with patients, reassuring them of their typically benign and transient nature.

## Author Contributions

**Saara Huoponen**: conceptualisation, methodology, investigation/data collection, formal analysis, writing and editing of the original draft. **Katrin Sisa**: conceptualisation, methodology, formal analysis, writing and editing of the original draft. **Tom Saari**: methodology, formal analysis, review of the original draft. **Markku Taittonen**: supervision, review and editing of the original draft. **Ulla Ahlmen‐Laiho**: supervision, conceptualisation, editing and review of the original draft.

## Conflicts of Interest

The authors declare no commercial conflicts of interest. This research was funded by a grant from the non‐profit Finnish Society of Anaesthesiologists.

### Peer Review

The peer review history for this article is available at https://publons.com/publon/10.1002/brb3.70227

## Data Availability

Data available on reasonable request from the authors.
